# The Effects of System Changes in Grazed Dairy Farmlet Trials on Greenhouse Gas Emissions

**DOI:** 10.3390/ani8120234

**Published:** 2018-12-07

**Authors:** Tony van der Weerden, Pierre Beukes, Cecile de Klein, Kathryn Hutchinson, Lydia Farrell, Tinke Stormink, Alvaro Romera, Dawn Dalley, Ross Monaghan, David Chapman, Kevin Macdonald, Robyn Dynes

**Affiliations:** 1 AgResearch, Invermay Agricultural Centre, Mosgiel 9053, New Zealand; cecile.deklein@agresearch.co.nz (C.d.K.); tinke.stormink@outlook.com (T.S.); ross.monaghan@agresearch.co.nz (R.M.); 2 DairyNZ Ltd., Private Bag 3221, Hamilton 3240, New Zealand; pierre.beukes@dairynz.co.nz (P.B.); lydia.farrell64@gmail.com (L.F.); alvaro.romera@dairynz.co.nz (A.R.); kevin.macdonald@xtra.co.nz (K.M.); 3 AgResearch, Grasslands Research Centre, Palmerston North 4410, New Zealand; kathryn.hutchinson@agresearch.co.nz; 4 DairyNZ Ltd., Canterbury Agricultural & Science Centre, Lincoln 7608, New Zealand; dawn.dalley@dairynz.co.nz (D.D.); david.chapman@dairynz.co.nz (D.C.); 5 AgResearch, Lincoln Research Centre, Lincoln 7674, New Zealand; robyn.dynes@agresearch.co.nz

**Keywords:** environmental modelling, pasture systems, nitrous oxide, methane emissions, nitrate leaching

## Abstract

**Simple Summary:**

Dairy farm system practices aimed at reducing nitrate leaching can also reduce emissions of the greenhouse gases methane and nitrous oxide. A study comparing ‘current’ and ‘improved’ grazed dairy system practices showed that ‘improved’ systems generally produced lower greenhouse gas emissions while milk production was maintained. The amount of feed eaten per hectare was the key driver of total greenhouse gas emissions per area, with ‘improved’ systems generally exhibiting lower total enteric methane and less N flowing through the herd.

**Abstract:**

An important challenge facing the New Zealand (NZ) dairy industry is development of production systems that can maintain or increase production and profitability, while reducing impacts on receiving environments including water and air. Using research ‘farmlets’ in Waikato, Canterbury, and Otago (32–200 animals per herd), we assessed if system changes aimed at reducing nitrate leaching can also reduce total greenhouse gas (GHG) emissions (methane and nitrous oxide) and emissions intensity (kg GHG per unit of product) by comparing current and potential ‘improved’ dairy systems. Annual average GHG emissions for each system were estimated for three or four years using calculations based on the New Zealand Agricultural Inventory Methodology, but included key farmlet-specific emission factors determined from regional experiments. Total annual GHG footprints ranged between 10,800 kg and 20,600 kg CO_2_e/ha, with emissions strongly related to the amount of feed eaten. Methane (CH_4_) represented 75% to 84% of the total GHG footprint across all modelled systems, with enteric CH_4_ from lactating cows grazing pasture being the major source. Excreta deposition onto paddocks was the largest source of nitrous oxide (N_2_O) emissions, representing 7–12% of the total GHG footprint for all systems. When total emissions were represented on an intensity basis, ‘improved’ systems are predicted to generally result in lower emissions intensity. The ‘improved’ systems had lower GHG footprints than the ‘current’ system, except for one of the ‘improved’ systems in Canterbury, which had a higher stocking rate. The lower feed supplies and associated lower stocking rates of the ‘improved’ systems were the key drivers of lower total GHG emissions in all three regions. ‘Improved’ systems designed to reduced N leaching generally also reduced GHG emissions.

## 1. Introduction

Agriculture is responsible for 47.9% of total greenhouse gas (GHG) emissions in New Zealand (NZ) but contributions to national emissions of methane (CH_4_) and nitrous oxide (N_2_O) are 86% and 95%, respectively [1]. Dairy farms primarily emit two GHG: (1) CH_4_ from enteric fermentation in the cow rumen, and (2) N_2_O arising mainly from denitrification of urinary nitrogen (N) in the soil and nitrogen fertiliser application. Methane emissions from dairy cattle have increased 130% from 1990 to 2015 [1]. In 2015, enteric CH_4_ was the major contributor (73%) while N_2_O from agricultural soils represented 21% of total GHG emissions from the agricultural sector [1]. The main drivers for this change are a doubling of the national dairy cow population since 1990 [2] and an increase in the application of synthetic N fertiliser (>600%) over this same period [1]. In New Zealand, dairy cows graze ryegrass-dominant pastures, of which perennial ryegrass (*Lolium perenne*) is the major species, with supplements (e.g., maize silage, barley grain) typically less than 100 g/kg of feed intake. Cows typically calve at the end of winter (i.e., July–September) and are milked for 8–10 months [3]. Similar pasture-based grazing systems for dairy cows are used in Australia [4,5] and Ireland [6].

An important challenge facing the NZ dairy industry, and globally, is to develop farm systems that can maintain or increase production (to meet increasing population demand) and profitability, while reducing impacts on receiving environments including water and air [7,8,9]. Various hypotheses have been advanced on changing dairy farm systems to reduce their environmental impact. Lowering stocking rate can result in more feed per cow, resulting in better-fed cows with more production per cow [3]. Fewer cows with better feed conversion efficiency could mean less feed is required for maintenance and more is converted into product. If cow genetic merit can be improved at the same time then these lower-stocked, well-managed systems can produce the same amount of product per hectare as higher-stocked systems [10]. The lower-stocked system will require less feed per area and, therefore, reduce the amount of N consumed and excreted by the herd. Nitrogen leaching will be reduced since the amount of urinary N deposited onto pasture is a major source of nitrate leaching [11]. Also, with lower feed intake from the smaller, more efficient herd it can be expected that the amount of enteric CH_4_ emitted will be reduced [3].

A reduction in N fertiliser use will usually reduce N leaching [12,13] and N_2_O losses from soils [14]. Less fertiliser will reduce the total amount of pasture grown, and also assist in a small reduction in the total N concentration of the herbage [4,15] and, therefore, reduce the amount of N flowing through the stock, and excreted as urinary and dung N. Reducing N fertiliser can be achieved through optimising its use, by targeting N application to pastures that have the greatest yield potential and to paddocks displaying signs of deficiency (yellowing and poor performance), rather than whole-farm N applications [16]. There is also the possibility of some compensation from less fertiliser through improved clover vigour and soil health resulting in greater natural N fixation [17,18].

Other strategies for reducing environmental impacts of dairy farm systems include improved reproductive performance of the herd, which results in less involuntary culling and lower replacement rates (reviewed in [19]). Replacements produce CH_4_ and urinary N without contributing to milk production [20]. Greater use of high energy/low N feed (grain or forage) will reduce total urinary N excreted through lowering N intake [21] and improve the energy intake when pasture growth or pasture quality is low. This strategy also dilutes the effect of excess crude protein supplied by the pasture [22]. Off-paddock facilities can be used to reduce N returns to pasture during periods of low N utilisation and in turn decrease the risk of N leaching in winter and spring [23]. In addition, off-paddock facilities protect wet pasture from treading damage [24,25]. 

Farmlet systems trials (Pastoral 21–Phase 2 (P21), [26,27]) were run over a five-season period from 2011 to 2016 with the aim of developing industry-accessible, adoptable, system-level solutions for profitably increasing production while reducing N leaching [7]. Four dairy regions were used to provide contrasting challenges to dairy production due to different soil types, climates and local management practices. ‘Improved’ dairy systems for each region were initially developed to improve water quality outcomes via strategic changes to the current system. Some of these changes were recognised to also deliver reduced emissions of GHG whilst maintaining or increasing milk production and profitability [3]. The key changes included:

1. Using fewer, higher producing, cows
2. Smaller N fertiliser inputs
3. Lower herd replacement rate
4. Greater use of high energy/low N feed
5. Using off-paddock facilities to reduce the time cows spend on pasture (or on forage crops).

These five components were used to design the P21 farmlet systems trials with all or some of them applied to the ‘improved’ system in each location. Here, we examine whether ‘improved’ dairy systems designed to reduced N leaching also reduce GHG emissions. The effects of these system changes, or ‘stacked mitigation options’, are evaluated for total GHG emissions and emissions intensity (kg GHG per kg milksolids) by comparing ‘current’ and ‘improved’ dairy systems in these locations. This analysis tests the hypothesis that system changes aimed at reducing nitrate leaching will also reduce total greenhouse gas emissions and emissions intensity (kg GHG per kg milksolids). As part of this study, we determined which of the five key system changes delivered the greatest benefit. 

## 2. Methodology

### 2.1. Farmlets

Relatively small-scale farms (farmlets ranging from 13 to 39 ha) were used to evaluate the system changes implemented in each of the three regions studied: the Waikato, Canterbury, and Otago (Table 1). These systems’ studies ran from 2011 to 2015 in the Waikato, from 2011 to 2014 in Canterbury and from 2012 to 2015 in Otago. 

In all systems, replacement stock were removed from the milking platform at birth and reared on support blocks, returning to the milking platform (pasture areas used for feeding milking cows) as rising 2-year old cows (~22 months) before calving. In the modelling analysis, the support was included in the inventory calculations to ensure analysis of a complete system. N fertiliser applications on these blocks were assumed as 100 kg/ha/year, based on expert opinion.

Details of the methodology used for estimating GHG emissions from the farmlets in all three regions can be found in the Supplementary File S1.

### 2.2. Waikato

Two farmlets (13 ha each) were established at Scott Farm, Hamilton, New Zealand (37°46′ S, 175°22′ E) in June 2011 [7]. One system represented a current Waikato farm system (‘current’), while the other employed technologies that might be required in improved farm systems (‘improved’) to reduce nitrate leaching. The ‘improved’ system was based on the concept of producing the same amount of milk per ha, but with the highest level of efficiency allowed by available technologies. 

The stocking and replacement rates were lower for the ‘improved’ system (Table 2) and dairy cows with higher genetic merit were used (breeding worth of $170 vs. $90, respectively; Table 2). Reducing the stocking rate (SR) increased the annual feed allowance per cow which, combined with the higher genetic merit of cows in the ‘improved’ system, led to increased kg MS/cow. All this translated into a reduced need for N input, as less feed was required (i.e., producing the same with less). 

An off-paddock facility was used in the ‘improved’ system where cows were removed from pasture onto a loafing pad for between 8 and 16 h daily from March until June (autumn until early winter). The loafing pad, also called a stand-off pad, was a plastic-lined area with wood chip bedding where cows could lie and where some of the dung and urine could be collected into the effluent system. The goal was to reduce N returns to pasture during a period of low N utilisation, thereby reducing N leaching risk during periods of drainage in winter/spring. All solid excreta deposited to the standoff pad was collected and stored until the following spring. A further goal was to protect wet pastures from treading damage. 

The ‘improved’ farmlet cows were offered up to 3 kg DM/cow/day of low-crude protein grain to improve their energy intake when pasture growth or pasture quality was low. This strategy also had the aim of diluting excess crude protein supplied by the pasture. The ‘current’ system used bought in pasture and maize silage when DM requirements could not be met from its pasture growth. 

Liquid effluent from the milking shed, collecting yard, and loafing pad was spread on the farmlets. Dung solids deposited in the milking shed and collecting yard were mechanically separated from the liquid phase for both systems. Emissions from these solids, and from solids captured by the loafing pad in the ‘improved’ system, were included in the GHG footprint calculations although these solids were exported from the farmlets to be spread in another location.

### 2.3. Canterbury

The Canterbury systems trial (43°38′ S, 172°28′ E) examined the effect of two ‘improved’ farm systems with contrasting stocking rates of 3.5 and 5 cows/ha for ‘improved(LOW)’ and ‘improved(HIGH)’, respectively (Table 2; [16]). The farmlet size was 8.25 ha milking platform (MP) plus 2 ha wintering crop (WC) and 6.75 ha MP plus 1 ha WC, respectively. 

The ‘improved(LOW)’ system used dairy cows with higher genetic merit than the ‘improved(HIGH)’ system (breeding worth of 140 vs. 133, respectively). 

A combination of ‘standard’ ryegrass/white clover pasture and ‘diverse’ pasture (containing chicory, plantain, ryegrass, and clover) was incorporated into the ‘improved(LOW)’ system, in contrast to the ‘improved(HIGH)’ system that solely relied on ‘standard’ ryegrass/white clover pasture. In the ‘improved(LOW)’ system, non-lactating cows were wintered on forage kale and oats silage, while the ‘improved(HIGH)’ system cows were wintered on fodder beet and pasture silage. Cow replacement rates were the same for the two systems. 

There was insufficient resourcing to include a ‘current’ system in Canterbury. However, a suitable farm nearby (Lincoln University Dairy Farm, ‘LUDF’) represented current Canterbury practices from 2011 to 2013, with a stocking rate of 4 cows/ha and non-lactating cows wintered on fodder beet [28]. Therefore, the LUDF was adopted as a representative ‘current’ system (Table 2), ensuring the methodology used for estimating the GHG footprint is consistent with the P21 farmlets. 

### 2.4. Otago

In Otago (46°17′ S, 169°43′ E), three farmlet systems were used, consisting of 110 cows each (Table 2). Firstly, a ‘current’ system (37 ha milking platform) adopted management practices typical of the region. Secondly, a ‘improved optimised’ (‘improved(OPT)’) system (39 ha) focused on improved cow feeding without the need for additional spending on costly farm infrastructure, and thirdly, a ‘improved duration-controlled grazing’ (‘improved(DCG)’) system (39 ha) that utilised an off-paddock facility (loose-housed deep litter animal shelter) for housing cows periodically during winter, spring, and autumn [27]. 

There were several key differences between the ‘current’ and the two ‘improved’ systems. Both the ‘improved(OPT)’ and ‘improved(DCG)’ systems included a lower replacement rate (18%) compared with 23% for the ‘current’ system, while N fertiliser applications were lower (42–73 kg N/ha/year) on the ’improved’ system milking platforms compared with 109 kg N/ha/year on the ‘current’ milking platform. In the ‘improved(OPT)’ system, pasture was supplemented with whole crop cereal silage during lactation. Short rotation (Italian) ryegrass pastures were also incorporated to better align pasture growth rates with cow feed demand. 

In addition, optimised grazing management of winter brassica crops along with allocation of more feed per cow during winter months were used to improve body condition score (BCS) relative to the ‘current’ herd. A key management goal of this farmlet was to ensure that cows calved later, and in better condition, onto higher pasture covers to decrease the reliance on N fertiliser and supplements and better match pasture growth with cow demand.

In the ‘improved(DCG)’ system cows were removed from pasture overnight (12 h) in spring and autumn during the milking season, when critical soil moisture thresholds and grazing times were reached, to protect pastures from damage and, for autumn grazing, reduce urinary nitrogen return to soils prior to winter. Shorter grazing times in spring led to relatively large amounts of pasture requiring conservation as silage on this farmlet; combined with the relatively large amounts of effluent returned to pasture (more details below), N fertiliser inputs were lower compared with the control farmlet and ranged between 63 and 83 kg N/ha/year. 

During winter, cows were housed full time in a loose-housed deep litter animal shelter with the aim of improving BCS relative to the ‘current’ herd through improved utilisation of feed energy. The shelter (767 m^2^) initially contained 300 m^3^ of woodchip bedding material, with another 150 m^3^ added midway through the winter period. Adjacent to the shelter was a feeding apron. Non-lactating cows were housed in the animal shelter fulltime during the winter months (June until mid-August; see Table S2) until calving commenced. 

Details of the manure management from the shelter use can be found in the Supplemental File. Briefly, liquid from excreta deposited in the animal shelter was collected in an effluent pond, whereas solid manure was removed from the shelter and stored prior to spreading onto the milking platform paddocks. Manure deposited onto the adjacent feeding apron was scraped and stored behind a weeping wall, with the liquid fraction contained in an effluent pond. Data collected by [29] were used for estimating the greenhouse gas emissions from the manure management.

### 2.5. Greenhouse Gas Emissions

Annual GHG emissions for each system were estimated for three (Canterbury and Otago) or four (Waikato) years, using calculations based on the New Zealand Agricultural Inventory methodology (NZAI; [1]). In brief, this methodology uses estimates of dry matter intake (DMI), N inputs, and N leaching losses, in combination with CH_4_ and N_2_O emission factors (EF). In this study a combination of NZAI emission factor values and CH_4_ and N_2_O emissions factors were used that were measured for key components of the milking platform or the wintering period for each system [30,31,32]. We used these targeted measurements to provide us with emission factor results for key components in the farmlets that we otherwise would not able to assess as the NZAI emission factors are not sufficiently disaggregated. For example, NZAI uses the same methane emission factor for all feeds and therefore cannot distinguish between different feed types. As some of the key changes in our ‘improved’ systems were related to different feed types, we used our targeted measurements to get more specific CH_4_ (and N_2_O) emission factors for these feed types. Similarly, as NZAI uses only one manure management system (anaerobic lagoons), we conducted targeted measurements of key components of the manure management system for the South Otago farmlet that used an off-paddock facility to ensure we could capture any difference in emissions as a result of the off-paddock facility. 

The GHG footprint boundary was limited to CH_4_ and N_2_O emissions, and excluded carbon dioxide (CO_2_) emissions from fertiliser manufacturing and use, fuel use, electricity use, and infrastructure construction. This ensured the footprint aligned with the boundaries of the NZAI. The footprint included both on-farm and off-farm sources of CH_4_ and N_2_O emissions. On-farm sources included enteric CH_4_ from the milking platform and wintering paddocks, N_2_O from soils receiving N inputs and CH_4_ and N_2_O emissions derived from manure management. Off-farm sources included N fertiliser use for producing pasture for replacement stock, N-excreta deposited by replacement stock, enteric CH_4_ from replacement stock and N fertiliser used for growing crops and supplements. 

In addition to farm-scale GHG footprints, key sources of emissions were separated to determine the impact of off-paddock facilities on GHG emissions by combining the sources of emissions that are influenced by the presence or absence of such a facility. These sources included (i) direct and indirect N_2_O emissions associated with excreta deposition onto paddocks, because removing cows from paddocks onto off-paddock facilities would directly influence the amount of excreta deposited onto paddocks, (ii) and N_2_O and CH_4_ emissions associated with manure collected, stored, and subsequently applied to land (i.e., manure management). Both off-paddock facilities included in this study were assessed, the loafing pad in Waikato and animal shelter in Otago. 

All CH_4_ and N_2_O emissions were converted to CO_2_-equivalent emissions using the 100-year time horizon global warming potentials of 25 kg CO_2_-equivalent per kg CH_4_ and 298 kg CO_2_-equivalent per kg N_2_O [33]. Greenhouse gas footprints for each system were calculated on an area basis (kg CO_2_-equivalent per milking platform hectare; kg CO_2_e/ha; Table 3) and intensity basis (kg CO_2_-equivalent per kg milksolids produced; kg CO_2_e/kg MS; Table 4).

## 3. Results and Discussion

### 3.1. CH_4_ Emissions

#### 3.1.1. CH_4_ Emissions per Area (kg CO_2_e/ha)

Methane represented between 75% and 84% of the total GHG footprint across all farmlet systems, with emissions ranging from 8892 kg CO_2_e/ha (‘improved(OPT)’, Otago) to 15,944 kg CO_2_e/ha (‘improved(HIGH)’, Canterbury) (Table 3). This broad range reflects contrasting feed supplies (sum of pasture production and supplements brought onto the farm) available to support stocking rate (SR) across systems ranging from 2.6 cows/ha in the Waikato ‘improved’ system to a substantially greater SR of 5.0 cows/ha in the ‘improved(HIGH)’ system in Canterbury (Table 2).

The majority of the emissions were from enteric fermentation by cows grazing pasture during lactation. Greater use of pasture as a feed source in Waikato compared against Canterbury and Otago explains the greater contribution of pasture-derived CH_4_ via enteric fermentation, representing 60–62% of the total GHG emissions (Table 3). Enteric CH_4_ from supplements was responsible for 8–10% of the total footprint in Waikato. In contrast, pasture-derived CH_4_ emissions for Canterbury and Otago represented 44% to 52% of the total GHG emissions per hectare while CH_4_ from supplements and winter forage crops collectively contributed 14–20% of the total GHG footprint (Table 3). 

In the Waikato, the ‘improved’ system produced 14% lower CH_4_ emissions per hectare compared with the ‘current’ system, while in Canterbury the ‘improved(LOW)’ system produced 22% lower CH_4_ emissions per hectare compared with the ‘improved(HIGH)’ system. These reductions were primarily driven by stocking rate, although the lower replacement rate in the ‘improved’ system in Waikato and lower CH_4_ from supplement and crop intake in the ‘improved(LOW)’ system in Canterbury also contributed to this reduction. In Otago, total CH_4_ emissions from the ‘improved(OPT)’ system were 6% lower than CH_4_ emissions from the ‘current’ system, primarily due to lower emissions via enteric fermentation during lactation. In contrast, the ‘improved(DCG)’ system emitted a similar amount of CH_4_ as the ‘current’ system, with reduced enteric fermentation on the milking platform and the lower emissions from replacement stock (via lower replacement rate) being balanced by a tripling of estimated CH_4_ emissions from manure storage and handling (Table 3).

#### 3.1.2. CH_4_ Emissions Intensity (kg CO_2_e/kg MilkSolids)

Milk production per unit area was relatively unaffected by farm system in the Waikato and Otago, whereas in Canterbury there was a substantial difference in kg MS/ha, with 1785 kg and 2335 kg and MS/ha being measured for the ‘improved(LOW)’ and ‘improved(HIGH)’ systems, respectively (Table 2). 

When emissions were expressed on an intensity basis, the data showed CH_4_ emissions from the Waikato ‘improved’ system were 10% lower than from the ‘current’ system (8.0 kg vs. 8.9 kg CO_2_e/kg MS, respectively; Table 4). This was due to the reduced feed requirements per unit of area, and thus enteric CH_4_ emission, enabled by the planned increase in production efficiency (less, more efficient, cows). In Canterbury, milk production in the ‘improved(LOW)’ system was 24% lower, similar to the reduction in CH_4_ emissions. As a result, CH_4_ emission intensities were similar for the two systems (6.9 vs. 6.8 kg CO_2_e/kg MS for the ‘improved(LOW)’ and ‘improved(HIGH)’ systems, respectively; Table 4). In Otago, CH_4_ emissions intensity was similar for all three systems, with the ‘current’, ‘improved(DCG)’ and ‘improved(OPT)’ systems producing 9.8 kg, 10.1 kg, and 9.6 kg CO_2_e/kg MS, respectively (Table 4).

### 3.2. N_2_O Emissions

#### 3.2.1. N_2_O Emissions per Area (kg CO_2_e/ha)

Nitrous oxide represented between 16 and 25% of the total GHG footprint across all farmlet systems, with emissions ranging from 1879 kg CO_2_e/ha (‘improved(DCG)’, Otago) to 4671 kg CO_2_e/ha (‘improved(HIGH)’, Canterbury), reflecting the SR, which ranged from 2.8 cows/ha (OPT and DCG, Otago) to 5.0 (‘improved(HIGH)’, Canterbury) (Table 3).

Excreta deposition onto paddocks was the largest source of N_2_O emissions, representing 9–12% of the total GHG footprint for all systems apart from ‘improved(DCG)’ in Otago, where this source represented only 7% of the total footprint. This latter system included removal of cows from paddocks when soils were wet, resulting in less excreta deposited onto soil. Nitrogen fertiliser use and emissions associated with replacement stock were the next most important sources of N_2_O emissions, where fertiliser represented between 1% and 4% of the total GHG footprint, while replacement stock represented 2% and 4% of the total GHG footprint.

#### 3.2.2. N_2_O Emissions Intensity (kg CO_2_e/kg MilkSolids)

In the Waikato, N_2_O emission intensity represented 1.8 kg CO_2_e/kg MS for the ‘improved’ system, which was 25% lower than the 2.4 kg CO_2_e/kg MS calculated for the ‘current’ system. The main driver of the reduction was less N entering the farm system in the form of N fertiliser and supplements. The reduction in pasture growth resulted in less pasture eaten for the farm system, thus reducing the excreta return (Table 4). Higher annual pasture allowance per cow, higher BW cows and longer lactations meant more milk was produced per cow in the ‘improved’ system. When combined, these factors resulted in lower emissions intensity i.e., kg CO_2_e/kg milksolids in the ‘improved’ system than for the ‘current’ system (Table 4).

In Otago, a 19% reduction in N_2_O emission intensity calculated for the ‘improved(DCG)’ system (from 2.4 to 2.0 kg CO_2_e/kg MS) was primarily driven by lower N fertiliser use, which reduced GHG intensity by 0.3 kg CO_2_e/kg MS. A further factor was the reduction in excreta deposition onto paddocks, achieved by removing cows for 12 h per day when soils were wet (autumn and spring) and full time in winter when cows were not lactating (Table S2). This management strategy accounted for a reduction of 0.5 kg CO_2_e/kg MS, which was slightly more than the increase in N_2_O emissions associated with additional manure management (+0.4 kg CO_2_e/kg MS). In Canterbury, emission intensity for the two ‘improved’ systems were similar, at 1.8–2.0 kg CO_2_e/kg MS.

### 3.3. Total GHG Footprint

#### 3.3.1. GHG Emissions per Area (kg CO_2_e/ha)

**Figure 1:**
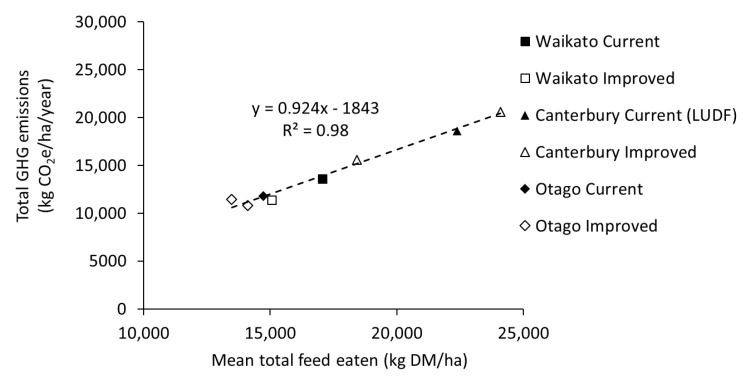
Relationship between mean total feed eaten (kg DM/ha) and total GHG emissions for ‘current’ and ‘improved’ systems trialled in three regions of New Zealand. Also included is the Canterbury ‘current’ system based on an updated analysis of the Lincoln University Dairy Farm (LUDF) system (P. Beukes, pers. comm.).

Total GHG footprints range between 10,792 kg and 13,610 kg CO_2_e/ha for the Waikato and Otago, with ‘improved’ systems producing a lower GHG footprint. This was particularly evident in the Waikato, where the reduction was 16%, while the reductions in footprint in the Otago farmlets were smaller, at 3% and 9% for the ‘improved(DCG)’ and ‘improved(OPT)’ systems, respectively. The emissions from the Canterbury systems were greater compared with other the regions, at 15,582 and 20,615 kg CO_2_e/ha for the ‘improved(LOW)’ and improved(HIGH)’ systems, respectively (Table 3). Total GHG emissions from the Canterbury ‘current’ system, which was based on data collated from the nearby Lincoln University Dairy Farm (SR of 4 cows/ha), were 18,628 kg CO_2_e/ha. The emissions from the ‘improved’ systems were either 11% higher (improved(HIGH)) or 16% lower (improved(LOW)) than from this ‘current’ system. Combining regions, total GHG emissions/ha were strongly related to the amount of feed eaten/ha (Figure 1).

The Canterbury systems had the largest contrast in feed eaten (18,400 vs. 24,100 kg DM/ha), resulting in the largest difference in GHG emissions per hectare (15,582 vs. 20,615 kg CO_2_e/ha; Table 3). Emissions from the LUDF ‘current’ system [28], were similar to the ‘improved(HIGH)’ system, at 18,628 kg CO_2_e/ha. However, this ‘current’ system produced 1870 kg MS/ha, which was substantially less than the milk production of the ‘improved(HIGH)’ system (2335 kg MS/ha; Table 2). 

**Figure 2:**
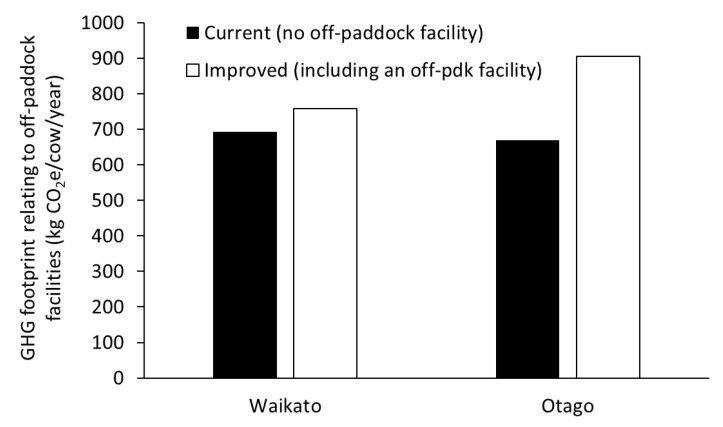
Effect of off-paddock facilities on net greenhouse gas emissions (kg CO_2_e/cow/year) associated with N_2_O from excreta deposition onto paddocks, and N_2_O and CH_4_ emissions from manure management.

Inclusion of off-paddock facilities in the Waikato and Otago ‘improved’ systems resulted in a decrease in excreta deposited onto paddocks and an increase in the amount of manure that required active management (see Table S4). To assess the impact of off-paddock facilities on GHG emissions, direct and indirect N_2_O emissions associated with excreta deposition onto paddocks and N_2_O and CH_4_ emissions associated with manure management were collated. Attempting to present these emissions on a per area basis can be difficult to interpret, given these facilities are farm structures; therefore, emissions have been calculated and presented as kg CO_2_e/cow/year. Our analysis showed that using an off-paddock facility results in a decrease in emissions per cow from excreta deposited onto paddocks, but this was more than offset by an increase in emissions per cow from manure management, resulting in a net increase in GHG emissions (Figure 2). The degree of the increase in emissions was dependent on the extent of the facility’s use. For instance, the loafing pad in Waikato increased manure/excreta-related GHG emissions by 10% while the off-paddock facility in Otago led to a 35% increase in associated emissions per cow (Figure 2).

In order to assess the potential impact of adopting ‘site-specific’ rather than ‘NZ-default’ EF values on the calculated GHG emissions, we compared our results to those calculated when adopting the EF values from the NZ inventory methodology (results not shown). Adopting the NZ-default EF values had very limited impact on the relative difference in emissions between ‘current’ and ‘improved’ systems. For Waikato, the reduction in emissions remained at 16%. For Canterbury, the increase in GHG emissions from ‘current to ‘improved(HIGH)’ changed from 7% (site-specific EFs) to 8% (NZ-default EFs). The decrease in GHG emissions from ‘current’ to ‘improved(LOW)’ changed from 18% to 19%. In Otago, using the NZ-default EF values did not impact on the relative difference between the ‘current’ and ‘improved(OPT)’, but it did slightly affect the result for ‘improved(DCG)’. When using the site-specific EF values that were based on experimental results, a 3% reduction in total GHG emissions was calculated. However, adopting the inventory approach resulted in a 1% reduction in GHG emissions between these two systems. This is most likely due to the fact that the NZ inventory methodology only includes the manure management system ‘anaerobic lagoons’, as this system is applicable to the vast majority of NZ dairy systems. However, given that the manure management system of the ‘improved(DCG)’ system is very different to the ‘anaerobic lagoon’ system, we believe the calculations based on the site-specific EF values are more accurate and realistic. Our study, therefore, represents the GHG footprint of dairy systems adopting ‘current’ and ‘improved’ management practices, estimated using the NZ inventory approach combined with site specific EF values where appropriate.

#### 3.3.2. GHG Emissions Intensity (kg CO_2_e/kg MilkSolids)

**Figure 3:**
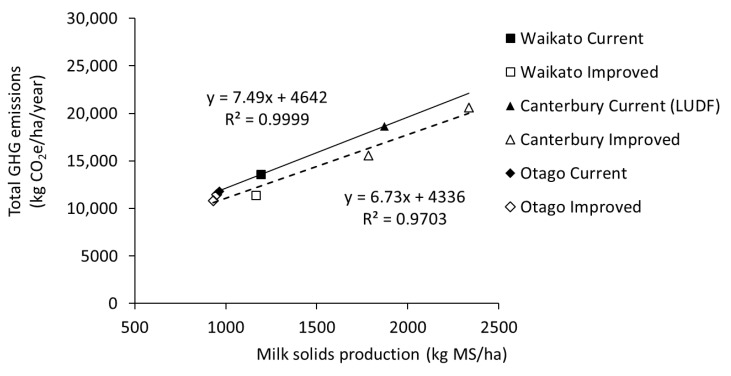
Relationship between milk production (kg MS/ha) and total GHG emissions for ‘current’ and ‘improved’ systems trialled in three regions of New Zealand. Also included is the Canterbury ‘current’ system based on an updated analysis of the Lincoln University Dairy Farm (LUDF) system (P. Beukes pers. comm.). There was no significant difference in the regression models.

When total emissions were represented on an intensity basis, emissions ranged from 8.7 kg to 12.3 kg CO_2_e/kg MS. The ‘improved’ systems in all three regions produced lower GHG emission intensities, with reductions of 13%, 11–12%, and 6% being calculated for ‘improved’ systems in Waikato, Canterbury, and Otago (OPT), when compared with the corresponding ‘current’ systems (Table 4). In most cases, the lower GHG intensities were largely a result of management practices such as reduced N fertiliser use and lower replacement rates lowering GHG emissions from the ‘improved’ system, because the difference in MS production between ‘improved’ and ‘current’ systems was small (Figure 3). For the ‘improved(HIGH)’ system in Canterbury, lower GHG intensities were largely a result of higher MS production, rather than reduced inputs. The ‘improved(DGC)’ system in Otago, based around standing cows off wet paddocks, and wintering cows in an animal shelter, produced a small reduction (2%) in the GHG emissions intensity due to increased emissions from manure management, as shown above. Across all regions, total GHG emissions/ha were strongly related to amount of milk produced/ha (Figure 3). Analysis of the data, determined by comparing a single regression model with a combined model that included system type as a treatment, showed there was no significant difference in the ‘current’ vs. ‘improved’ systems (*p* > 0.05). The lack of significance was possibly due to the relatively small dataset, as the results showed lower GHG emissions for the same MS production from the ‘improved’ systems compared to the ‘current’ systems.

We compared our GHG emission intensity results with Gerber et al. [34], which presents N_2_O and CH_4_ emissions intensities from dairy cattle systems in 155 countries. For the comparison, we converted our results from kg MS to kg of fat and protein corrected milk (FPCM; [34]). The NZ dairy cattle systems modelled in our study produced between 4200 and 6700 kg FPCM per year, with CH_4_ emissions of between 0.6 and 0.8 kg CO_2_e per kg FPCM and N_2_O emissions of between 0.1 and 0.2 kg CO_2_e per kg FPCM. Our results therefore compared well with those presented by Gerber et al. [34] for moderate to high intensity production systems.

### 3.4. General Discussion

Lower total GHG emissions, albeit not significant, were demonstrated in the ‘improved’ systems. Farm system trials are conducted at relatively large scales, making it challenging to replicate system treatments. Consequently, we have not been able to demonstrate ‘significantly’ lower GHG emissions from the ‘improved’ systems. However, our results do suggest that system changes aimed at reducing nitrate leaching can also reduce total greenhouse gas emissions and emissions intensity. The amount of feed eaten per ha was the key driver of total GHG emissions per area (Figure 1). Pasture-based dairy farming systems in the temperate environment of New Zealand have evolved to match seasonal pasture supply to the feed demand. The profitability and productivity of these systems is driven by stocking rate (cows/ha) which enables very high pasture utilisation and reasonable production per cow [35,36]. Both targeted nitrogen fertiliser application and supplement use contribute to feed supply (per hectare) and enable flexibility in stocking rate and profitability for many farming businesses. The amount of feed eaten per ha has an overriding effect on GHG emissions per ha because enteric CH_4_ from lactating cows is the major contributor to GHG emissions, as is the case with other pasture-based dairy systems [8,37]. Lower stocking rates are necessary when lower N inputs result in lower feed supply per ha (a function of N inputs including direct N fertiliser use for on-farm pasture production and off-farm supplement production). It has been shown that lower N inputs reduce farm-gate N surplus and thus the potential risk of N losses to the environment e.g., [7,38]. Others have also observed this relationship through measurements [39,40] and modelling [41]. Limiting the amount of N brought into a farm system as N fertiliser and supplements will reduce N intake and excretion by the herd, depositing less urinary N onto paddocks, and ultimately reducing N_2_O emissions. When combined with having cows with greater genetic merit (in the Waikato study), an increased feed conversion efficiency meant less feed was required for the herd for maintenance and more feed converted into product, resulting in lower feed requirements but similar milk production per hectare. Previous modelling [3,42,43] showed that the combination of reduced stocking rates and high genetic merit cows consistently reduced total GHG emissions and emissions intensity, but with inconsistent impacts on milk production. The Waikato ‘improved’ system presents an example of a future vision, with minimal sacrifice of production per hectare (2%) whilst demonstrating reductions in GHG emissions (by 16% and 13% when expressed on an area or emissions intensity basis, respectively) and N leaching (by 40–50%; [7]). As for the Waikato, the Canterbury and Otago ‘improved’ systems also required less fertiliser N input, which lowered N_2_O emissions. The ‘improved (OPT)’ system in Otago also produced a lower GHG emission intensity (6% reduction), however there was also a small decrease in MS production (3% reduction). Estimated N leaching for this system was reduced by 23% [27], providing another example of how GHG emission and N loss reductions could be achieved, albeit not as substantially as found for the Waikato study. 

Others have also demonstrated reduced on-farm GHG emissions with less N fertiliser use (e.g., [44]). A combination of reduced N fertiliser use and lower stocking rates has been shown to have the largest impact on GHG emissions [45,46]. Using a Life Cycle Assessment method, Basset-Mens et al. [47] assessed the eco-efficiency of three contrasting New Zealand dairy systems. These researchers concluded that GHG emissions per area and per unit product (i.e., intensity-based) was lowest with the least intensive system, where no N fertiliser was used and cow stocking rate was 2.3 per ha compared with more intensive systems supporting 3.0 and 5.2 cows per ha. Potential eutrophication of waterways was projected to follow a similar pattern, with the least intensive system having the smallest potential for impact on waterways [47]. 

Manure management impacted heavily on the GHG footprint associated with the ‘improved(DCG)’ system, resulting in a footprint similar to that of the Otago ‘current’ system (Table 4). This negated any benefits achieved from removing cows off wet paddocks. 

Previous work by Garnsworthy [48] predicted that improving fertility levels and breeding management in dairy cows, and therefore reducing the number of heifer replacements required, could reduce methane emissions at a herd level by 10% to 11%. In a modelling study by Beukes et al. [3], they estimated the contribution of the reduced replacement rate strategy to GHG reductions to be in the order of 5%. In the two regions of this study where replacement rates decreased from ‘current’ to ‘improved’ (Waikato from 22% to 18%, and Otago from 23% to 18%), we estimated a reduction of 3% to 4% in total GHG emissions from the ‘current’ system. The farmlet trials in the Waikato and Otago demonstrated the merit of lower herd replacement rates from the current New Zealand average of 22–23% [2] to c. 18% as an option for making modest reductions in total GHG emissions.

One of the Canterbury systems included a contrast of feed types, with the ‘current’ and ‘improved(HIGH)’ systems relying on standard ryegrass/white clover pasture swards on the milking platform while non-lactating cows were wintered on fodder beet and pasture silage. In contrast, 40% of the milking platform in the ‘improved(LOW)’ system consisted of diverse pasture containing chicory, plantain, ryegrass, and clover, with non-lactating cows wintered on forage kale and oat silage. Although the diverse pasture was modelled to produce lower N_2_O EF_3_ values for deposited urine [30], the emission intensities associated with total N_2_O loss from dung and urine did not differ between the two systems (Table 4). This was partly due to the ‘improved(LOW)’ system including kale in the winter period, which had a higher EF_3_ value than the fodder beet crop grazed in the ‘current’ and ‘improved(HIGH)’ systems. 

Given the potential benefits of fodder beet over kale, for both CH_4_ and N_2_O emissions, and the similar amounts of silage consumed (see Supplement A), substituting kale for fodder beet in the ‘improved’ system, may result in even lower total GHG emissions for the ‘improved’ system. A similar suggestion was presented by Chapman et al. [16] for N leaching. These authors concluded that if the Canterbury ‘improved(LOW)’ system incorporated a fodder beet winter crop (rather than kale), N leaching could be reduced by 25–28%.

The Otago farmlet systems included the use of palm kernel expeller (PKE) in year 3 (Table S1; Supplement A). When averaged over the three years of the study, the use of PKE represented 1.9%, 0.5%, and 1.7% of the dry matter intake for the CON, OPT, and DCG systems, respectively. There have been recent concerns linking consequences of indigenous deforestation with palm oil production, which has placed pressure on restricting the use of PKE on NZ dairy farms [49]. Deforestation results in additional GHG emissions through land use change, among other impacts. While our study has focused solely on biological emissions (i.e., N_2_O and CH_4_ only) from dairy production, we have estimated the increase in total GHG emissions for the Otago systems when the carbon (C) footprint associated with the use of PKE is included. A recent study suggests a C footprint of 0.506 kg CO_2_-equivalents per kg PKE DM used on NZ dairy farms [50]—this value includes emissions due to land use change. Based on this value, the additional C footprint from the use of PKE in the CON, OPT, and DCG systems in Otago averaged over three years was estimated to be 49, 12, and 43 kg CO_2_e/ha/year, respectively. Compared to the biological emission-based footprint (Table 3), the inclusion of the LCA-based C footprint associated with PKE use represents an additional 0.4%, 0.1%, and 0.4%, respectively. This additional footprint does not change the emission intensity values shown in Table 4, as the increase was less than <0.1 kg CO_2_e/kg MS. 

The New Zealand dairy industry aims to identify dairy systems that can maintain or increase production while reducing impacts on receiving environments including water and air [9]. As such, the ‘improved’ systems designed in this study included a package of measures that incorporated the best available knowledge to reduce impacts on water whilst maintaining or increasing productivity [7,16,27]. Improved systems will be more attractive to farmers if they deliver additional benefits such as reduced impact on water quality, or if a reduction in milk production is not associated with a reduction in profitability. Across the three regions, the implementation of stacked mitigation options aimed at reducing N leaching may have changed the relationship between MS and GHG emissions per hectare. While the analysis across regions showed no significant difference in the regression models describing milk production and GHG emissions for ‘current’ and ‘improved’ systems, probably due to the relatively small dataset, it does suggest a reduction in emissions intensity may be possible. 

Our modelling suggests, for the systems examined here, total GHG emissions could be reduced by between 4% and 16%. Given the New Zealand dairy industry plan to contribute to meeting this nation’s 2030 emissions reduction target of 30% below 2005 levels [51], our modelled reductions are relatively modest. These reductions are based on currently available management options and research into additional agricultural mitigation options is continuing to be an important focus for New Zealand [48]. This includes research into developing low-methane animals, methane vaccines and inhibitors, low GHG feeds, and novel nitrification inhibitors [52,53,54]. Increasingly ‘improved’ dairy systems modelled here, and future strategies identified through the current research efforts, will be essential options for farmers to meet social and regulatory requirements in New Zealand. Profitability and cost effectiveness of the ‘improved’ systems modelled here are yet to be explored. 

## 4. Conclusions

Our analysis indicates that system changes aimed at reducing nitrate leaching can also reduce total greenhouse gas emissions and emissions intensity. The reduced feed supplies and associated lower stocking rates of the ‘improved’ farmlet systems evaluated here were the key drivers of lower total GHG emissions in all three regions. The main effects of these improved farmlet attributes were smaller total enteric methane emissions and less N flowing through the herd, which lowered N excretion and, therefore, direct and indirect N_2_O losses. A system with fewer cows with greater genetic merit contributed to greater milk production per cow per lactation, less N leaching, and lower GHG emission intensities. Smaller but important contributions to lowering emissions were made by dietary changes, e.g., introducing low-protein grain supplements, cereal silages, fodder beet winter feed, herb-containing ryegrass pastures, and by lowering herd replacement rates. Off-paddock facilities contributed to protecting wet soils and reducing N leaching, but resulted in pollution swapping and increased total GHG emissions per cow. Our results suggest that total GHG emissions can be reduced through lower-stocked systems, where individual cow performance is optimised through better feeding of high genetic merit animals to compensate for the lower stocking rates. 

## Figures and Tables

**Table 1:** ‘Improved’ system changes applied to farmlet system trials in Waikato, Canterbury, and Otago, New Zealand.

Region	Fewer, Higher Producing, Cows	Reduced N Fertiliser Inputs	Reduced Herd Replacement Rate	Greater Use of High Energy/Low N Feed	Off-Paddock Facilities
Waikato	✓	✓	✓	✓	✓
Canterbury	✓	✓		✓	
Otago		✓	✓		✓

**Table 2:** Key management features of ‘current’ and ‘improved’ systems in Canterbury, Waikato, and Otago for assessing differences in, and key drivers of, GHG emissions from dairy systems. Estimated N leaching losses are also included [7,16,27]. HIGH and LOW = high and low stocking rate; OPT = optimised feeding and DCG = duration controlled grazing. See text for further descriptions of each systems.

	Waikato		Canterbury			Otago		
System Features	Current	Improved	LUDF (2011–2013)	Improved (HIGH)	Improved (LOW)	Current	Improved (OPT)	Improved (DCG)
Milking platform								
Stocking rate (cows/ha)	3.2	2.6	4.0	5.0	3.5	3.0	2.8	2.8
Cow genetic merit ($BW ^#^)	90	170	115	133	140	109	105	104
N fertiliser (kg N/ha/year)	137	52	345	311	158	109	42	73
Replacement rate (%)	22	18	23	23	23	23	18	18
High energy/low N feed	0	0.24 (Grain t DM/cow/year)	N/A	N/A	40% diverse pasture	N/A	N/A	N/A
Stand-off/housing	No	Yes	No	No	No	No	No	Yes
Milksolids production *								
(kg MS/ha/year)	1193	1163	1870	2335	1785	964	931	949
(kg MS/cow/year)	373	447	468	467	510	329	333	334
Wintering								
Winter feed	On platform	On platform	Fodder beet + Pasture silage	Fodder beet + Pasture silage	Kale + Oat silage	Kale	Kale	N/A
N fertiliser (kg N/ha/year)	N/A	N/A	150	200	307	200	200	N/A
Total dairy system N loss								
N leaching (kg N/ha/year)	54	31	54	55	58	18 ^^^	13 ^^^	11 ^^^

LUDF: Lincoln University Dairy Farm; N/A: not applicable; ^#^ Breeding worth, $ (May 2011); * Fat + Protein, measured in P21 study; ^^^ assessed as estimated N leaching [27].

**Table 3:** Total emissions (kg carbon dioxide equivalents per hectare of the milking platform (kg CO_2_e/ha MP) calculated for nitrous oxide (N_2_O) and methane (CH_4_) for improved farm systems tested on small-scale farms in three regions in New Zealand (Waikato, Canterbury, and Otago). HIGH and LOW = high and low stocking rate; OPT = optimised feeding and DCG = duration controlled grazing. See text for further descriptions of each systems. Note: may not sum to total due to rounding.

Source		Waikato		Canterbury			Otago		
		Current	Improved	LUDF (2011–2013)	Improved (HIGH)	Improved (LOW)	Current	Improved (OPT)	Improved (DCG)
		3.2 Cows/ha	2.6 Cows/ha	4 Cows/ha	5 Cows/ha	3.5 Cows/ha	3 Cows/ha	2.8 Cows/ha	2.8 Cows/ha
N_2_O	Urine + dung	1602	1012	2110	2309	1519	1229	1108	760
	Fertiliser	390	150	1108	990	665	448	270	220
	Manure mgmt	99	390	254	31	21	11	9	387
	NH_3_ volatilised	320	288	632	442	308	250	202	220
	NO_3_ leached	217	162	247	223	241	86	64	44
	Replacement stock	285	189	346	676	460	329	246	249
	Total N_2_O	2913	2191	4697	4671	3213	2353	1900	1879
CH_4_	Ent Ferm. Pasture	8131	7044	9195	8965	8085	5752	5330	5493
	Ent Ferm. Supplement + Winter crop	1079	1081	2601	4120	2235	1982	2175	1976
	Ent Ferm. Replacement stock	1363	906	1541	2226	1558	1308	976	987
	Manure mgmt	124	183	594	633	490	433	411	1127
	Total CH_4_	10,697	9214	13,931	15,944	12,368	9475	8892	9582
Total CO_2_e (kg)		13,610	11,405	18,628	20,615	15,582	11,827	10,792	11,461

**Table 4:** Emissions intensity (kg carbon dioxide equivalents per milksolids (kg CO_2_e/kg MS)) calculated for nitrous oxide (N_2_O) and methane (CH_4_) for current and improved farm systems tested on small-scale farms in three regions in New Zealand (Waikato, Canterbury, and Otago). HIGH and LOW = high and low stocking rate; OPT = optimised feeding and DCG = duration controlled grazing. See text for further descriptions of each systems. Note: may not sum to total due to rounding.

Source		Waikato		Canterbury			Otago		
		Current	Improved	LUDF (2011–2013)	Improved (HIGH)	Improved (LOW)	Current	Improved (OPT)	Improved (DCG)
		1193 kg MS/ha MP	1164 kg MS/ha MP	1870 kg MS/ha MP	2335 kg MS/ha MP	1785 kg MS/ha MP	964 kg MS/ha MP	931 kg MS/ha MP	949 kg MS/ha MP
N_2_O	Urine + dung	1.3	0.9	1.1	1.0	0.9	1.3	1.2	0.8
	Fertiliser	0.3	0.1	0.6	0.4	0.4	0.5	0.3	0.2
	Manure mgmt	0.1	0.3	0.1	<0.1	<0.1	0.0	0.0	0.4
	NH_3_ volatilised	0.3	0.2	0.3	0.2	0.2	0.3	0.2	0.2
	NO_3_ leached	0.2	0.1	0.1	0.1	0.1	0.1	0.1	<0.1
	Replacement stock	0.2	0.2	0.2	0.3	0.3	0.3	0.3	0.3
	Total N_2_O	2.4	1.8	2.5	2.0	1.8	2.4	2.0	2.0
CH_4_	Ent Ferm. Pasture	6.8	6.1	4.9	3.8	4.5	6.0	5.7	5.8
	Ent Ferm. Supplement + Winter crop	0.9	0.9	1.4	1.8	1.3	2.1	2.3	2.1
	Ent Ferm. Replacement stock	1.1	0.8	0.8	1.0	0.9	1.4	1.0	1.0
	Manure mgmt	0.1	0.2	0.3	0.3	0.3	0.4	0.4	1.2
	Total CH_4_	8.9	8.0	7.4	6.8	6.9	9.8	9.6	10.1
Total CO_2_e (kg)		11.3	9.8	9.8	8.8	8.7	12.3	11.6	12.1

## Supplementary Materials

The following are available online at http://www.mdpi.com/2076-2615/8/12/234/s1, Supplementary File S1.
