# Supplementary material for: The Effects of System Changes in Grazed Dairy Farmlet Trials on Greenhouse Gas Emissions
Source: Animals (Basel). 2018 Dec 7;8(12):234. doi: 10.3390/ani8120234 (PMC6316583; doi:10.3390/ani8120234)
Supplement: Supplementary file 1 [file animals-08-00234-s001.pdf]

Supplementary File 1

Methods by region

In some cases, methods differed between regions. In some regions we had more detailed data available and were thus able to use a more tailored approach to estimate GHG emissions. In the other regions we used a more generic approach that fitted the available data. In all cases we used the best data that was available for each region to calculate GHG emissions.

Methane emissions

Total methane emissions for each system were calculated from the sum of the methane emissions from enteric fermentation, manure management, dung deposition onto paddocks and replacement stock.

Methane from enteric fermentation

Methane emissions from enteric fermentation from each system were calculated based on estimated DMI (2) for the different feeds used (on both the milking platform and wintering block) and CH_4 yields (g CH_4/kg DMI) for these feeds. We used the New Zealand Agricultural Inventory methodology (NZAI; [1]), as follows:

Enteric methane (g CH_4/cow/day) = Estimated dry matter intake (kg DMI/cow/day) x CH_4 yield (g CH_4/kg DMI) ***(S1)***

Estimating Dry Matter Intake

Dry matter intake of cows grazing pastures was estimated from the difference between pre- and post-grazing pasture covers, measured one week apart, in Waikato and Canterbury. Over this period there would be no effective growth. In Waikato, visual estimates of pasture covers were done every three days and calibrated weekly against pasture cuts. In Canterbury, pre and post-grazing measurements were made using a rising plate pasture meter [2], with 'clicks' being converted to DM using the 'all seasons' equation ([3]; Equation S2).

DM (kg/ha) = number of clicks x 140 + 500 **(S2)**

In Otago, there was no regular assessment of post grazing pasture covers to allow DMI to be estimated, therefore dry matter intake of cows grazing pastures was estimated from the difference between daily metabolisable energy (ME) intake required for measured milk production and the ME supplied by supplements for each cow (Equation S3).

DM intake (kg/cow/day) = (Daily energy requirement (MJ ME/cow/day) – Total energy supplied via supplements (MJ ME/cow/day))/Metabolisable energy of pasture (MJ ME/kg DM) ***(S3)***

where 'daily energy requirement' was estimated based on the daily MS, 'total energy supplied via supplements' was calculated for each supplement using DM offered (based on either measured crop biomass or tonnage supplied multiplied by DM content) x % utilisation (assumed to be 85%; [4]) x ME content. The ME content was based on either analysis of collected feed samples (pasture silage/baleage, whole crop cereal silage (WCCS), kale) or published data (turnips, palm kernel extract (PKE); [4]). 'Metabolisable energy of pasture' was estimated to be 12 MJ ME/kg DM, based on the analysis of 33 pasture samples using near infra-red spectroscopy (NIRS: ARL laboratories, Ravensdown, New Zealand; [5]) collected over the three seasons (range of 11.0 and 12.7 MJ ME/kg DM).

For the winter crop fed in Canterbury and Otago, a daily DM allowance was set for animals on each crop, and the size of the grazed area determined based on this allowance and the estimated crop biomass. Crop biomass was determined from weekly or fortnightly (Canterbury and Otago, respectively) quadrat cuts of the winter crop harvested to ground level (five 1x1m quadrats in the kale crop and three 2x2 m quadrats in the fodder beet crop. After grazing in Canterbury, further quadrat cuts were taken to estimate utilisation of the grazed crop. For the forage kale crop an average utilisation of 85% was measured in Canterbury, based on these measurements the same utilisation rate was assumed for the Otago forage kale. The utilisation of the fodder beet crop was 100%.

In Otago, pasture baleage DMI for the ‘current’ and ‘improved(OPT)’ non-lactation wintering period was estimated from daily allowances and an assumed utilisation rate of 85%. For the ‘improved(DCG)’ non-lactation wintering period, pasture silage DMI was estimated from daily allowances and an assumed utilisation rate of 95% [6]. Grain and silage DMI was estimated from daily allowances and a measured utilisation rate in both Waikato and Canterbury. Average annual DM intake estimates from the different feeds are given in Table S1.

Methane yield from dry matter intake

For the Waikato and Otago systems the NZAI default CH_4 yield from feed for New Zealand livestock, 21.6 g CH_4/kg DMI, was used for all feed types [7]. In Canterbury, contrasting winter forage species provided an opportunity for targeted CH_4 measurements using GreenFeed emissions measurement units [8] were conducted to estimate CH_4 yields from ryegrass pasture and the two winter forage crops used (kale and fodder beet). The measured CH_4 yields were 22.3, 23.3 and 16.5 g CH_4/kg DMI for ryegrass pasture, kale and fodder beet, respectively [8]. CH_4 yields for other feed types (i.e. grain and silage) were assumed to be the same as that measured for the Canterbury ryegrass pasture (22.3 g CH_4/kg DMI). Although inclusion of grain in the diet can result in a lower methane yield [9], a recent meta-analysis showed that this only occurs when the proportion of grain in the diet is at least 40% [10]. As the proportions of grain in the diet on the milking platforms of the ‘improved(LOW)’ and ‘improved (HIGH)’ systems in Canterbury were 2 and 9%, respectively, we assumed that the methane yield of the grain was the same as for ryegrass pasture.

Methane from manure management

Animal shelter use in the RES system in Otago was based on data from Chrystal *et al*. [11]. The activity data for the manure management system was based on information collated for the 2014 wintering and shoulder seasons, as data for earlier years was not available due to a delay in the animal shelter construction. Excreta input was estimated on the basis of animal

shelter usage (Table S2), cow DMI and feed N content, corrected and N exported as milk protein.

For the winter of 2014, 110 non-lactating cows consumed ca. 100 t DM pasture silage DM over a 70-day period. The silage had an N content of 1.5%, resulting in an N take of 0.190 kg N/cow/day. After accounting for N use for maintenance the estimated N excretion of each cow present in the barn was 0.185 kg N/cow/day.

For the shoulder seasons of 2014, a total of 2989 cow.days were spent in the barn, where the daily dietary intake of 10 kg DM pasture and 5 kg DM pasture silage with N contents of 3.0% and 2.1%, respectively, resulted in a daily excreta production of 0.40 kg N/cow/day after correcting for N removed as milk.

The total amount of N excreted in the barn during the shoulder and winter periods was estimated to be 2400 kg N. Based on the average N content of the feeding apron manure and a volume of 63 m^3 of excreta being scraped from the pad to the weeping wall [11], it was estimated that 300 kg N-excreta, or 12% of the total barn excreta production, was deposited onto the pad. The weeping wall had a volume of 50 m^3 and was assumed to be empty at the start of winter (1 June). We also assumed the weeping wall was used for 6 months, after which the solids were removed and applied to land.

The barn was emptied on 15 July, 1 September and early October: the total volume of manure (excreta + woodchip) removed on 15 July and 1 September was 550 m^3, with an average N content of 0.48% [11]. New woodchip was added for the spring and following autumn use. To provide a fair assessment of a single year's use, we have estimated the spring manure volume and N load (excreta + woodchip) based on the use over this period and the associated use and manure generation in the preceding autumn/winter period. Conversion of woodchip volume to weight was estimated by assuming a bulk density of 0.4 t/m^3 (as measured from a nearby barn with similar woodchip; [12]).

Our analysis suggested a total woodchip volume of 500 m^3 was used for the shoulder and winter seasons in 2014. The N content of fresh woodchip was 0.04% [13], resulting in an N load of 135 kg woodchip-N. The amount of excreta-N deposited onto the woodchip in the barn was estimated at 2115 kg N based on the difference between the estimated total N excretion (2409 kg N) and the load deposited onto the feeding apron (294 kg N). The total N load (excreta + woodchip) in the barn was estimated at 2250 kg N (2115 + 135 kg N), of which 58 kg N drained out of the barn into the effluent pond (246 m^3 of liquid, with an N content of 0.23 kg N/m^3; [11]).

Methane from dung deposition onto paddocks

All regions required an estimate of CH_4 emissions from dung; this required an estimate of the mass of CH_4 multiplied by the emission factor of CH_4 from dung. The mass of dung (Faecal dry matter; FDM) produced by animals was determined from dry matter intake (DMI, kg/cow; equation S4).

$$FDM\ (kg/cow) = DMI\ (kg\ DM/cow) \times (100\text{-}DMD)/100 \qquad (S4)$$

Where FDM is the faecal dry matter (kg FDM/cow), DMI is Dry Matter Intake (kg DM/cow) and DMD is Dry Matter Digestibility (%). The total amount of FDM produced on the milking platform during lactation was disaggregated between pasture (0.95) and effluent pond (0.05) [1]. However, it is important to note that in the Waikato system, solids were separated from liquid, with the solids treated similarly to loafing pad solids (i.e. stored prior to land application), while the liquid fraction entering the pond. We, therefore, assumed no carbon entered the Waikato pond. Urine and dung deposited onto farm tracks was assumed to have the same emissions profile as that deposited on pasture.

Dry matter digestibility (%) was estimated from the metabolisible energy (ME) values (expressed as megajoules of metabolisable energy per kilogram DM (MJ ME/kg DM)) for all feeds using equation S5, which was derived from equations reported by the Australian Feeding Standards for Ruminants [14].

$$DMD\% = ME/0.152 + 0.9 \quad (S5)$$

Dung deposited onto pastoral soils is a source for CH_4 emissions, which were estimated following the New Zealand inventory approach [1]. CH_4 emissions from deposited dung were calculated based on FDM output and CH_4 emission factor:

$$Direct\ CH_4\ (kg\ CH_4/year) = FDM\text{-}pdk\ \times CH_{4\ DUNG} \quad (S6)$$

Where FDM-pdk is the estimated amount of faecal dry matter deposited onto paddocks (kg FDM/year) and $CH_{4\ DUNG}$ is the CH_4 emission factor for FDM as dung (0.00098 kg CH_4/kg FDM; [1]). This value was also used to estimate the yield of CH_4 from dung spread as solids from the milking parlour and loafing pad for the Waikato farmlet systems (which was estimated to be 24% of dung produced on the milking platform, 5% from the parlour and 19% from the loafing pad, based on the number of days and hours spent on the pad).

Methane emissions from effluent and solid manure storage were estimated following the New Zealand inventory approach used for effluent ponds, and adapting this for solid manures. This involved estimating the amount of FDM stored and calculating an appropriate EF value for each form of manure stored. The forms of manure included (i) effluent in ponds, (ii) solid manure from separated solids, loafing pad solids and animal shelter solids, and (iii) slurries stored in weeping walls.

The amount of FDM stored as effluent in Canterbury and Otago was based on the disaggregation between paddock and effluent pond. For Waikato, all of the separated solids from the effluent were stored as solids. It was also estimated that 19% of the paddock FDM was deposited onto loafing pads in this region. It was assumed that the separated solids from effluent and solids from the loafing pad would be similar in composition, classed as 'solid storage' based on the IPCC guidelines [15].

In Otago, we estimated FDM production from cows in the animal shelter by adjusting the total FDM production by the number of days cows spent in the animal shelter (Table S2).

Total FDM production was partitioned into IPCC manure descriptions, with manure deposited and stored in the animal shelter equating to 'solid storage', manure deposited on the feeding apron and subsequently stored in the weeping wall equating to 'liquid/slurry' and effluent stored in ponds as 'anaerobic lagoon'. The partitioning, based on activity data [11], suggested there was 21.0 t FDM stored in the barn, 2.9 t FDM stored in the weeping wall and 6.8 t FDM stored in the effluent pond in 2014.

The CH_4 emission factors for manure management (effluent ponds, solid manures and slurries) were derived from the IPCC ([15], equation 10.23 and 10.24) (equation S7, [1]).

$$CH_{4\ MM} = ((100 - ASH)/100) \times B_o \times 0.67\ kg/m^3 \times MCF/100 \qquad (S7)$$

where $CH_{4\ MM}$ is the CH_4 emission factor for manure management (kg CH_4/kg FDM), ASH is the ash content of the feed calculated as a percentage of the dry matter feed intake (8%, [15,16]), B_o is the maximum methane producing capacity for dairy cattle manure (0.24 m^3 CH_4/kg VS excreted), 0.67 is the conversion factor for m^3 CH_4 to kg CH_4 and MCF is the methane conversion factor for each type of manure management system. Variables used for B_o and MCF and the resulting $CH_{4\ SM}$ emission factors for manure management are shown in Table S3.

Methane from replacement stock

The CH_4 emissions from the excreta and enteric fermentation of replacement stock grazed off the milking platform were included in the system emissions estimation calculations. No DMI measurements were taken for these animals therefore it was estimated that with a mature live weight of 500 kg they required 3,738 kg DM/head from 3 to 22 months of age [4]. Methane emissions were estimated using the same method as for the cows (Equations S1-S3). Enteric CH_4 emissions were calculated for each animal then multiplied by the stocking and replacement rates of the systems to estimate the enteric CH_4 emissions from replacement stock per ha of the milking platform.

Nitrous oxide emissions

Each system's total nitrous oxide emissions were assessed from the different components of the farm system (soils, manure management, pre-farm gate and replacement stock), as described below:

Estimating excreta N production

For Waikato and Otago, excreta N production (Nex) was estimated from total DMI and N content of the different feed types, corrected for N exported as milk protein, based on the approach used by the NZAI [1] and assumes that 1 kg protein contains 0.16 g N [17] (equation S8):

Total Nex (kg N/cow/year) = (DM intake (kg DMI/cow/year) x N in diet (kg N/kg DMI) – Milk yield (kg/cow/year) x protein content (kg protein/kg milk) x 0.16 (kg N/kg protein))

(S8)

Pasture DM and crude protein (CP) content of pasture was assessed monthly from July to May by collecting and analysing pasture samples, with CP converted to N content. A similar approach was adopted for pasture silage, cereal silage and winter kale crops, with fewer samples collected across each season.

For Canterbury, a slightly different methodology was used. Monthly measurements of the N content and creatinine content in the urine and the animal body weight were used in an equation that assumes a constant creatinine clearance factor of 21.9 mg/per kg live weight [18]. Total N excretion per cow was estimated based on the estimated total amount of urine-N excreted per unit of body weight and the monthly average animal live weight. Total Nex-dung was then estimated for all farmlets from Nex-urine (Table S4) and the N content of the dry matter based on the equations used in the NZAI methodology:

The proportion of N excreted as urine (%) = 10.5 x N content in diet (%) + 34.4 **(S9)**

The proportion of N excreted as dung (%) = 100 - the proportion of N excreted as urine **(S10)**

Inputs of N fertiliser were the actual N fertiliser rates applied to the farmlets (Table S4).

Estimating manure volume and N content

The N applied to paddocks as solid manure from the off-paddock facilities was estimated using an N balance approach, where the proportion of N deposited on the off-paddock facility was corrected for associated gaseous N emissions and removal of N as liquid effluent. For the 'improved(DCG)' system in Otago, the total amount of N excreted in the shelter during the shoulder and winter periods was estimated to be 2,400 kg N. Total gaseous N losses were estimated at 200 kg N, or 8% of deposited excreta-N, based on the amount of Nex and emission factors for N_2O, NH_3 and N_2 (Table S5). The N_2O emission factor ($Frac_{LossMS\text{-}N2}$) was estimated by means of an N balance between stored manure, N emitted as NH_3 and N_2O and N removed in liquid and solid forms. A total volume of 800 m^3 liquid as drainage from the barn and weeping wall, with an N load of 352 kg N, entered the effluent pond. Ammonia loss during effluent storage (35% of total N content lost as NH_3; Table S5) reduced the N load applied to pasture to 215 kg N. Table S5 provides a summary of the various N inputs onto soils for the dairy systems across all three regions.

Nitrous oxide from soils

The N_2O emissions from soils were assessed for key sources that collectively contribute to the GHG emissions either as direct or indirect N_2O emissions through inventory-type calculations based on N input and N_2O emission factors:

Direct N_2O (kg N/year)

$$= (Nex\text{-}urine \; x \; EF_{3\,urine}) + (Nex\text{-}dung \; x \; EF_{3\,dung}) + (Nurea \; x \; EF_{1urea}) + (Neff \; x \; EF_{1FDE}) + (Nsolids \; x \; EF_{1SM}) \quad (S11)$$

Where $N_{ex\text{-}urine}$, $N_{ex\text{-}dung}$, N_{urea}, N_{eff} and N_{solids} are the amounts of urine N, dung N, urea fertiliser N, effluent N and solid manure N deposited or applied to soils, respectively; and EF_{3urine}, EF_{3dung}, EF_{1urea}, EF_{1FDE} and EF_{1SM} are the N_2O emission factors for urine N, dung N, urea fertiliser N, farm dairy effluent N and solid manure N, respectively (Table S6).

In addition, indirect N_2O emissions from all systems were assessed from NH_3 volatilised and NO_3 leached

$$Indirect\ N_2O\ (kg\ N/year) = (NH_3\ volatilised\ x\ EF_4) + (NO_3\ leached\ x\ EF_5) \quad (S12)$$

with

$$NH_3\ volatilised = (Nex\text{-}urine + Nex\text{-}dung + Nfert + Neff + Nsolids)\ x\ Frac_{Gas} \quad (S13)$$

and

NO_3 leached (kg N/ha/yr) = as assessed in the P21 programme using in-field measurements (Waikato) or the nutrient budget model Overseer (Table 1) *Error! Reference source not found.****for Canterbury [19,20], or calculated as the potentially leachable N for Otago [21]).***

Where, NH_3 volatilised and NO_3 leached are the amounts of ammonia volatilisation and nitrate leaching respectively; EF_4 and EF_5 are the N_2O emission factors for volatilised and leached N, respectively; and $Frac_{GAS}$ is the NZ value for NH_3 volatilisation (Table S6).

For farm systems that include off-paddock facilities (i.e loafing pad in Waikato and animal shelter in Otago), solid manure (Nsolids) was included. Whereas, this source was omitted for farm systems where cows were not removed from pasture or crop.

Nitrous oxide from manure management

Direct and indirect N_2O emissions from animal excreta deposited and stored in manure management systems, including FDE ponds, were included in the inventory calculations. Direct emissions were estimated by multiplying the amount of N stored in various forms through the manure management system by the respective emission factor.

Direct N_2O from manure management (kg N/year)

$$= (Nex\ LP \times EF_{3LP}) + (Nex\ SSM \times EF_{3SSM}) + (Nex\ WW \times EF_{3WW}) \quad \textbf{(S14)}$$

Where $N_{ex\text{-}LP}$, $N_{ex\text{-}SSM}$ and $N_{ex\text{-}WW}$ are the amounts of excreta-N (N_{ex}) deposited/stored on or in loafing pads, animal shelters and weeping walls, while EF_{3LP}, EF_{3SSM} and EF_{3WW} refers to the N_2O emission factors for manure stored in or on loafing pads, animal shelters, weeping walls and effluent ponds, respectively (Table S5). We excluded direct N_2O emisison from effluent ponds (termed 'anaerobic lagoons' in the IPCC guidelines) due to IPCC default emission factors for N_2O equating to 0% [15].

In addition, indirect N_2O emissions via NH_3 volatilised from the various forms of manure stored were included.

Indirect N_2O from manure management(kg N/year) = (NH_3 volatilised x EF_4)   (S15)

$$\text{with } NH_3 \text{ volatilised} = (Nex\text{-}LP \times Frac_{GasMS\text{-}LP}) + (Nex\ SSM \times Frac_{GasMS\text{-}SSM}) + (Nex\ WW \times Frac_{GasMS\text{-}WW}) + (Neff \times Frac_{GasMS\text{-}AL}) \quad \textbf{(S16)}$$

Where $N_{ex\text{-}LP}$, $N_{ex\text{-}SSM}$, $N_{ex\text{-}WW}$ and N_{eff} are the amounts of excreta-N (N_{ex}) deposited/stored on or in loafing pads, animal shelters, weeping walls and effluent ponds, while $FracGas_{MS\text{-}LP}$, $FracGas_{MS\text{-}SSM}$, $FracGas_{MS\text{-}WW}$ and $FracGas_{MS\text{-}AL}$ refers to the fraction of N volatilised as NH_3 from each respective manure source.

The value of each direct emission factor (EF_3) and fraction of N volatilised ($Frac_{Gas}$) are shown in Table S6. Nitrate leaching was not included as an indirect source of N_2O from

manure management as it was assumed there was no loss of liquid from the storage systems [1].

Pre-farm gate

The pre-farm gate emissions were based on the amounts of supplement brought onto the milking platform. The direct and indirect N_2O emissions from fertiliser used to grow the imported supplements were estimated. For pasture silage the amount of fertiliser N was estimated using the ratio of pasture grown to N fertiliser use on the platform, while for maize silage and maize grain fertiliser N requirements were estimated based on data from Pioneer [22].

Nitrous oxide from replacement stock

The direct and indirect N_2O emissions from replacement stock grazed off the milking platform were included in the system emissions estimation calculations. A value of 3,738 kg DMI/head over the first 22 months [4] was used for all systems. N_2O emissions were estimated using the same method as for the cows. Values were calculated per hectare of the milking platform based on the stocking rate multiplied by the replacement rate for each system. Excreta N production (Equation S8) was estimated from total DMI and N content of the feed but was not corrected for N exported as milk protein. Partitioning of total excreta N production to urine and dung was calculated using equations S9 and S10. Nitrogen fertiliser inputs were estimated as 100 kg N/ha/year to grow pasture for replacement stock, with direct and indirect emissions calculated as per the milking platform. Direct and indirect N_2O from replacement stock excreta and indirect N_2O from N leaching from excreta and N fertiliser calculated as per the milking platform. No excreta was deposited in manure management systems for the replacement stock.

N_2O and NH_3 emission factors and fractions

Waikato

New Zealand default emission factors were used for all calculations except for N_{ex} deposited on the loafing pad [23] (Table S5 and Table S6).

Canterbury

New Zealand default emission factors and fractions were used for all calculations, except for EF_3-urine for the milking platform, i.e. for cows on standard pasture ('improved(HIGH)' system) vs cows on a combination of standard and diverse pasture ('improved(LOW)' system); and for EF_3-urine for the wintering crop, i.e. cows on fodder beet ('improved(HIGH)') vs cows on kale ('improved(LOW)'). The values for these emission factors were obtained from targeted N_2O emission measurements [24]; Table S6 provides an overview of these and other relevant emission factors and fractions used for estimating N_2O emissions for the two systems.

Otago

Nitrous oxide and NH_3 emission factors and fractions used in the GHG footprint calculation of the manure management system associated with the off-paddock facility, weeping wall and effluent pond are shown in Table S5. Emission factors for the solid manure and weeping wall manure were based on recent research [12,25].

New Zealand default emission factors and fractions were used for the majority of calculations. There were two exceptions; the first relates to the grazing of winter forage crops where high stocking rates for short-durations lead to severe soil compaction and deformation during wet winter months [26], thereby changing soil conditions that have a significant influence on N_2O emissions from deposited urine. The second relates to emissions following solid manure application to paddocks: this type of manure has a lower available N pool compared with farm dairy effluent, thereby potentially lowering EF values when based on the total N content of the manure. Alternative EF values based on recent research [12,25,27,28]

were used for urine deposited onto winter kale paddocks and solid manure applied to paddocks ($EF_{1\text{-}SM}$).

References

1. Ministry for the Environment. New Zealand's Greenhouse Gas Inventory 1990-2015. Publication number: ME 1309, Ministry for the Environment, ISSN 1179-223X (electronic), 2017; pp. 502; Available online: http://www.mfe.govt.nz/node/23304/.

2. Reynolds, R. Feed wedges: what are they and how can they help you? Proceedings of the South Island Dairy Event Conference, 2007, Lincoln, New Zealand, pp. 110-117.

3. DairyNZ pasture assessment. Available online: https://www.dairynz.co.nz/feed/pasture-management/assessing-and-allocating-pasture/pasture-assessment/ (Accessed 7 December 2017).

4. DairyNZ. Facts and figures for New Zealand Dairy Farmers. 2nd ed, 2017. DairyNZ, Hamilton, New Zealand.

5. Dalley, D.E.; Geddes, T. Pasture growth and quality on Southland and Otago dairy farms. *Pr NZ Grassl Assoc* **2012**, *74*, 237-242.

6. Dalley, D.E. (DairyNZ, Lincoln, New Zealand). Personal communication, 2017.

7. Clark, H.; Brookes, I.; Walcroft, A. Enteric Methane Emissions from New Zealand Ruminants 1990–2001 Calculated Using an IPCC Tier 2 Approach. Report prepared for the Ministry of Agriculture and Forestry by AgResearch and Massey University, 2003. Ministry of Agriculture and Forestry, Wellington, New Zealand.

8. Jonker, A.; Scobie, D.; Dynes, R.; Edwards, G.; de Klein, C.; Hague, H.; McAuliffe, R.; Taylor, A.; Knight, T.; Waghorn, G. Feeding fodder beet decreased methane emissions from dry and lactating dairy cows in pastoral systems. *Anim Prod Sci* **2017**, *74*, 145-147.

9. Beauchemin, K.A.; McGinn, S.M. Methane emissions from feedlot cattle fed barley or corn diets. *J Anim Sci* **2005**, *83*, 653-661.

10. Moate, P.J.; Deighton, M.H.; William, S.R.O.; Pryce, J.E.; Hayes, B.J.; Jacobs, J.L.; Eckard, R.J.; Hannah, M.C.; Wales, W.J. Reducing the carbon footprint of Australian milk production by mitigation of enteric methane emissions. *Anim Prod Sci* **2016**, http://dx.doi.org/10.1071/AN15222.

11. Chrystal, J.; Monaghan, R.; Hedley, M.; Horne, D. Volumes and nutrient concentrations of effluent products generated from a loose-housed wintering barn with woodchip bedding. In *Integrated nutrient and water management for sustainable farming*. L.D. Currie; M.J. Hedley Eds. Occasional Report No. 29, 2016. Fertilizer and Lime Research Centre, Massey University, Palmerston North, New Zealand. Available online: http://flrc.massey.ac.nz/publications.html.

12. van der Weerden, T.J.; Rutherford, A.J. Greenhouse gas emission factors for stored and land applied manure collected from indoor cows – Milestone report. NZAGRC-PGGRC Integrated farm systems programme 2014-2017 (Objective 8.2.4). Prepared for NZAGRC, 2016, pp. 29.

13. Chrystal, J. (AgResearch, Invermay, New Zealand). Personal communication, 2017.

14. Corbett, J.L.; Freer, M.; Hennessy, D.W.; Hodge, R.W.; Kellaway, R.C.; McMeniman, N.P.; Nolan, J.V. Feeding Standards for Australian Livestock Ruminants, 1990. Standing Committee on Agriculture, CSIRO Publishing, Melbourne, Australia.

15. IPCC. Emissions from livestock and manure management. In *Guidelines for National Greenhouse Gas Inventories*, Chapter 10. Intergovernmental Panel on Climate Change, 2006, pp. 87. Available online: http://www.ipcc-nggip.iges.or.jp/public/2006gl/pdf/4_Volume4/V4_10_Ch10_Livestock.pdf.

16. Dämmgen, U.; Amon, B.; Gyldenkærne, S.; Hutchings, N.; Klausing, H.K.; Haenel, H-D.; Rösemann, C. Reassessment of the calculation procedure for the volatile solids excretion rates of cattle and pigs in the Austrian, Danish and German agricultural emission inventories. Landbauforschung Volkenrode 61, 115-126.

17. Pierce, W.C.; Haenisch, E.L. *Quantitative Analysis*; John Wiley & Sons: London, UK, 1947.

18. Pacheco, D.; Burke, J.L.; Death, A.F.; Cosgrove, G.P. Comparison of models for estimation of urinary nitrogen excretion from dairy cows fed fresh forages. In *Meeting the Challenges for Pasture-Based Dairying*. 3rd Dairy Science Symposium, 18th-20th September 2007, University of Melbourne, Victoria, Australia. pp. 417-422.

19. Beukes, P.C.; Romera, A.J.; Gregorini, P.; Macdonald, K.A.; Glassey, C.B.; Shepherd, M.A. The performance of an efficient dairy system using a combination of nitrogen leaching mitigation strategies in a variable climate. *Sci Total Environ* **2017**, *599-600*, 1791-1801.

20. Chapman, D.; Edwards, G.; Dalley, D.; Cameron, K.; Di, H.; Bryant, R.; Romera, A.; Clement, A.; Malcolm, B.; Curtis, J. Nitrogen leaching, production and profit of irrigated dairy systems using either low or high inputs of fertiliser and feed: The Pastoral 21 experience in Canterbury. In *Science and policy: nutrient management challenges for the next generation.* L. D. Currie; M. J. Hedley Eds. Occasional Report No. 30. Fertilizer and Lime Research Centre, Massey University, Palmerston North, New Zealand, 2017, http://flrc.massey.ac.nz/publications.html.

21. Shepherd, M.; Hedley, M.; MacDonald, K.; Chapman, D.; Monaghan, R.; Dalley, D.; Cosgrove, G.; Houlbrooke, D.; Beukes, P. A summary of key messages arising from the Pastoral 21 Research Programme. In *Science and policy: nutrient management challenges for the next generation*. L.D. Currie, M.J. Hedley Eds. Occasional Report

No. 30. Fertilizer and Lime Research Centre, Massey University, Palmerston North, New Zealand, 2017. http://flrc.massey.ac.nz/publications.html.

22. Pioneer. Available online: http://www.pioneer.co.nz/maize-silage/product-information/silage-technical-insights/fertiliser-application-for-maize-crops.html. (Accessed 20 December 2016).

23. Luo, J.; Saggar, S. Nitrous oxide and methane emissions from a dairy farm stand-off pad. *Anim Prod Sci* **2008**, *48*, 179-182.

24. Di, H.J.; Cameron, K.C.; Podolyan, A.; Edwards, G.R.; de Klein, C.A.M.; Dynes, R.; Woods, R. The potential of using alternative pastures, forage crops and gibberellic acid to mitigate nitrous oxide emissions. *J Soils Sediments* **2016**, *16*, 2252-2262.

25. van der Weerden, T.J.; Luo, J.; Dexter, M.; Rutherford, A.J. Nitrous oxide, ammonia and methane emissions from dairy cow manure during storage and after application to pasture. *New Zeal J Agr Res* **2014**, *57*, 354-369.

26. Laurenson, S.; Houlbrooke, D.J.; Beukes, P.C. Assessing the production and economic benefits from preventing cows grazing on wet soils in New Zealand. *J Sci Food Agri* **2016**, *96*, 4584-4593.

27. van der Weerden, T.J.; Styles, T.M. Reducing nitrous oxide emissions from grazed winter forage crops. *Pr NZ Grassl Assoc* **2012**, *74*, 57-62.

28. van der Weerden, T.J.; Styles, T.M.; Rutherford, A.J.; de Klein, C.A.M.; Dynes, R. Nitrous oxide emissions from cattle urine deposited onto soil supporting a winter forage kale crop. *New Zeal J Agr Res* **2017**, *60*, 119-130.

Table S1. Estimated annual average dry matter intake (DMI; kg DM/cow/year) and methane yields (g CH_4 kg^{-1} DM intake) for feeds used in the 'current' and 'improved' systems in Canterbury, Waikato and Otago. HIGH and LOW = high and low stocking rate; OPT = optimised feeding and DCG = duration controlled grazing. See text for further descriptions of each systems. For CH_4 yield, values are New Zealand default yields unless otherwise indicated in bold.

	Waikato		Canterbury		Otago		
	Current	Improved	Improved(HIGH)	Improved(LOW)	Current	Improved(OPT)	Improved(DCG)
Milking platform							
Pasture	4,705	5,017	3,920	4,625	3,315	3,321	3,386
Pasture silage	515	458	640	370	283	63	377
Grain	N/A	238 (Maize)	475	110	N/A	N/A	N/A
Maize silage	109	74	N/A	N/A	N/A	N/A	N/A
Cereal silage	N/A	N/A	N/A	N/A	151	376	68
Turnips	N/A	N/A	N/A	N/A	106	N/A	N/A
PKE	N/A	N/A	N/A	N/A	42	10	37
CH_4 yield	21.6	21.6	**22.3**[A]	**22.3** [A]	21.6	21.6	21.6
Wintering block							
Fodder beet	N/A	N/A	455	N/A	N/A	N/A	N/A

CH_4 yield	N/A	N/A	**16.5** [A]	N/A	N/A	N/A	N/A
Forage kale	N/A	N/A	N/A	515	600	829	N/A
CH_4 yield	N/A	N/A	N/A	**23.3** [A]	21.6	21.6	N/A
Pasture silage	N/A	N/A	380	N/A	22	5	945
Oat silage	N/A	N/A	N/A	390	N/A	N/A	N/A
Pasture baleage	N/A	N/A	N/A	N/A	391	436	N/A
CH_4 yield	N/A	N/A	**22.3** [A]	**22.3** [A]	21.6	21.6	21.6
Overall total Dry Matter Intake							
	5,330	5,787	5,870	6,010	4,910	5,040	4,813

N/A: not applicable

[A] [8]

Table S2. Animal shelter use by cows in the ‘improved(DCG)’ farmlet, Otago 2014 (adapted from [11]). DCG = duration controlled grazing. See text for a further description of this system.

	Autumn (14 April – 26 May)	Winter (5 June – 31 August)	Spring (16 September – 30 October)
Number of days use	43	70	45
Hours/day	12	24	12
Cow hours in animal shelter	44,472	186,408	27,252

Table S3. Calculation of $CH_{4\ MM}$ EF values for effluent and manure storage (terms in brackets relate to IPCC descriptors).

	ASH (%)	B_o (m^3 CH_4/kg VS excreted)	MCF (%)	$CH_{4\ MM}$ (kg CH_4/kg FDM)
Effluent ponds ('Anaerobic lagoons')	8	0.24	74	0.109
Separated solids, loafing pad manure and animal shelter manure ('Solid storage')	8	0.24	4	0.006
Weeping wall ('Liquid/slurry')	8	0.24	27	0.040

Table S4. Annual average urine ($N_{ex\text{-}urine}$) and dung ($N_{ex\text{-}dung}$) excretion, farm dairy effluent (N_{eff}) and solid manure (N_{solids}) applications to land (kg N/ha/year) for the milking platform and winter crop blocks of each 'current' and 'improved' system in Canterbury, Waikato and Otago. HIGH and LOW = high and low stocking rate; OPT = optimised feeding and DCG = duration controlled grazing. See text for further descriptions of each systems.

	Waikato		Canterbury		Otago		
	Current	Improved	Improved(HIGH)	Improved(LOW)	Current	Improved(OPT)	Improved(DCG)
Milking platform							
$N_{ex\text{-}urine}$	318	252	362	245	176	145	143
$N_{ex\text{-}dung}$	145	124	166	110	93	83	78
N_{eff}	16	86	N/A	N/A	14	12	19
N_{solids}	7	69	N/A	N/A	N/A	N/A	52
Winter crop							
$N_{ex\text{-}urine}$	N/A	N/A	307	158	133	139	N/A
$N_{ex\text{-}dung}$	N/A	N/A	204	129	106	115	N/A

Table S5. N_2O emission factors (EF in % N_2O-N) and NH_3 volatilisation loss fractions (Frac in % NH_3-N) for manure management systems. Values are New Zealand default emission factors unless otherwise indicated in bold.

Component of calculation	Code	Waikato	Canterbury	Otago
N_2O emission factor for excreta deposited onto loafing pad.	EF_{3LP}	**0.01**[A]	N/A	N/A
N_2O emission factor for solid storage of manure.	EF_{3SSM}	**0.5**[B]	N/A	**1.2**[C]
N_2O emission factor for manure stored in weeping wall.	EF_{3WW}	N/A	N/A	**0.1**[D]
Fraction of excreta N from loafing pad lost as NH_3	$Frac_{GasMS\text{-}LP}$	10	N/A	N/A
Fraction of manure N from solid storage lost as NH_3	$Frac_{GasMS\text{-}SSM}$	N/A	N/A	**1.4**[E]
Fraction of manure N from weeping wall lost as NH_3	$Frac_{GasMS\text{-}WW}$	N/A	N/A	**5.7**[F]
Fraction of effluent N lost as NH_3 during storage	$Frac_{GasMS\text{-}AL}$	35	35	35
N_2O emission factor for NH_3 volatilisation.	EF_4	1.0	1.0	1.0

[A] [23]; [B] [15]; [C, E] mean values calculated from [12] and [25]; [D, F] [25]

Table S6. N_2O emission factors (EF in % N_2O-N) and NH_3 volatilisation loss fractions (Frac in % NH_3-N) for N deposited or applied to soil. Values are New Zealand default emission factors unless otherwise indicated in bold. HIGH and LOW = high and low stocking rate. See text for further descriptions of each systems.

		Waikato	Canterbury				Otago	
		Milking platform	Milking platform		Winter crop		Milking platform	Winter crop
Component of calculation	Code		Improved (HIGH)	Improved (LOW)	Improved (HIGH)	Improved (LOW)		
N_2O emission factor for urine	EF_{3urine}	1.0	**1.12**[A]	**1.12**[A](ryegrass)<br>**0.84**[A] (diverse)	**0.85**[A]	**1.1**[A]	1.0	**2.0**[B]
N_2O emission factor for dung	EF_{3dung}	0.25	0.25		0.25		0.25	0.25
N_2O emission factor for urea fertiliser	EF_{1urea}	0.59	0.59		0.59		0.59	0.59
N_2O emission factor for farm dairy effluent	EF_{1FDE}	0.25					0.25	0.25
N_2O emission factor for solid manure applied to land	EF_{1SM}	1.0	N/A		N/A		**0.02**[C]	N/A
N_2O emission factor for N leached	EF_5	0.75	0.75		0.75		0.75	0.75
Fraction of N inputs lost through NH_3 volatilisation	$Frac_{Gas}$	10	10		10		10	10
N_2O emission factor for NH_3 volatilisation	EF_4	1.0	1.0		1.0		1.0	1.0

[A] [24]; [B] mean value calculated from [25] and [27]; [C] mean value calculated from [12] and [25].
